# Monthly Summary

**Published:** 1883-11

**Authors:** 


### MONTHLY SUMMARY.

---

SOMETHING ABOUT SMOOTHNESS OF DENTAL PLATES.—All kinds of theories have been advanced to account for the various effects of rubber plates, plates of celluloid or metal on the mucous membrane of the mouth. Several factors contribute to these disagreeable effects: The non-conductivity of the materials, the silver and mercury they contain, but far more still, as it seems to me, the defective smoothness of rubber and celluloid plates which renders them so hurtful. We are all familar with the fact that, whenever two parts in the body touch and move upon each other, they are of extreme smoothness; in fact, so smooth, that it is very difficult for us to produce anything similar. I only draw attention to the cartilage of the bones and the outer surface of the eye ball. With people who are forced to use artificial eyes—though made of glass, the hardest and smoothest substance we can produce by a simple process—have become too rough for the eyelids, after only two years' wear. The liquids of the eye, the ammoniacial and saline secretions together with the continuous movements of the eye-lids, produce a superficial abrasion and roughness, even of this very hard and smooth material, so as to render it an injurious body to the eye-lids. The very same thing is important in dental plates. Why do plates lined with metal prove less injurious than simple rubber plates?

When at the Cincinnati Convention last year, Dr. Robinson, of Jackson, Michigan, mentioned the good results he obtained from rubber plates lined with his soft, fibrous foil. I thought that the heat conducting theory could not be made to agree with this fact. I was convinced from this and other facts that there was more than the heat conducting power which comes into play, and this factor I think is to be found in the defective smoothness of the ordinary rubber plates. Metal can be polishod much smoother than rubber or celluloid. Therefore, make your plates very smooth, either by covering them with some metallic lining, or by plating them with silver, gold or anything of the kind, or by using metal altogether.—*N. E. Journal of Dentistry.*

---

A SINGULAR CASE.—In a private letter recently from Prof. Watlinti, of Ypsillanti, Michigan, is described a novel affection. Of it, he says: " It has proved to be a very interesting case.

I have had it on hand for some time, and I am likely to, I fear for some time to come. It is in the mouth of a lady about forty-five years of age, and presents an opening through the gum of the lower jaw, at about the point where the third molar should be. The opening is from one-third to one-half an inch in diameter, and the passage within nearly as large. The canal passes backward and upward, just on the outside of the jaw, and back into the cheek, to a point nearly opposite the middle of the ear, where there is a large cavity in the location of the parotid gland. It has the appearance of the breaking down and wasting away of this gland. When I began treating it quite large pieces of yellowish material, resembling gland substance came away, but so soft and broken down that the microscope revealed little or nothing of structural character. The opening and canal has existed for two or three years, the general health is not disturbed by it, there is not much pain at present, nor has there ever been severe pain. There is no appreciable discharge from the opening. I am greatly interested to know just the nature of the case. What is the matter? Is it possible for the parotid gland to break down and waste away in that manner? The duct of steno is closed. I have consulted with some of our physicians and they are as much in the dark as to the cause of the trouble and its nature as I am."

I recently saw this case, and found it about as described above. The health of the patient is good, and the part affected is evidently improving by the manner of treatment employed, which is occasionally washing with mild disinfectant and antiseptic preparations, no general treatment has seemed to be required.

Is it possible that the parotid gland could be thus destroyed without producing more disturbance than has existed in this case.—*Dental Register.*

---

BONE DEGENERATION IN THE INSANE.—in the Section of Psycology at the late meeting of the British Medical Association (*British Medical Journal*, September), Dr. Joseph Wiglesworth read a paper on this subject, which concludes as follows:

"As before mentioned, the number of cases included in this communication being small, the induction reached can only be an imperfect one ; nevertheless, for the purposes of discussion, it is advisabie that certain conclusions should be drawn, which may be thus formulated :

"1. The ribs of lunatics are perfectly healthy in a minority of cases.

"2 The majority present some slight degree of change, which consists in a slight thinning of the external layer of compact bone, and slight enlargement of the Haversian canals ; but that these changes are in general merely trival, and to be correlated with the general failure of nutrition so common in insanity, or with the presence of a wasting disease such as phthisis, or with the advent of old age, or it may be with a combination of all these ; these cases possess, therefore, a general, not a local significance.

"3. In a minority of cases, provisionally estimated at ten per cent., clear and precise lesions are found, produced by considerable internal absorption, which renders the bone very porous and brittle, and brings it under the category of the condition known as osteoporous. The proportion of cases in which this affection occurs being thus considered to be much higher amongst insane than amongst sane individuals, it would appear to have some casual connection with insanity, of the nature of which we are as yet ignorant."—*Medical and Surgical Reporter*.

---

HOW DO PARASITES PRODUCE DISEASE?—Dr. George Sutton thus answers this question in a paper read before the Indiana State Medical Society (*Cinn. Lam. and Clinic*, July 21, 1883):

1. By producing poisonous compounds, as septicæmia, from the decomposition of tissue.

2. By changing the composition of the blood, as seen in typhus fever, yellow fever, plague, etc.

3. By the destruction of tissues in syphilis, cancer, etc.

4. By irritation of the nervous system, as convulsions arising from worms, etc.

5. By consumption of nutriment, consequently draining the vital forces from the system, as when 20,000,000 trichina invade the human body.

6. By thrombus of small arteries, as seen in the anthrax, etc., etc.

7. By local irritation, producing total inflammation, as we see in scabies and ring-worm, etc.

---

TRANSPLATION OF MUSCLE IN MAN.—Helferich (*Archiv. f. Klm. Chirurgie* B. xviii., p. 562) reports a case in which, as a result of the removal of fibro-sarcoma from the arm of a woman aged thirty-six, the whole upper half of the biceps, with the exception of a thin strand at is outer part, was extirpated. Into the cavity which was left he promptly introduced a large fragment of the biceps from the leg of the dog. The cut surfaces were carefully brought together with sutures, as little injury as possible being done to the parts. The transplanted muscle was much more voluminous than the original portion, and was long after the operation distinctly perceptible to the touch. Electric experiments instituted about three months after the operation showed that their biceps reacted perfectly naturally to both kinds of current. The high point of stimulation situated at the place of section of the musculo cutaneous nerve was, however, absent. The movements at the elbow-joints were almost normal.—*Medical Gazette.*

